# Cellular heterogeneity and multicellular mechanisms in the pathogenesis of late-life depression: insights from single-cell and spatial multi-omics

**DOI:** 10.3389/fimmu.2026.1944035

**Published:** 2026-09-15

**Authors:** Yao Gao, Ze-Kun Li, Han-Ni Li, Jian-Zhen Hu, Xin-Zhe Du, Xin Yan, Sha Liu

**Affiliations:** 1 Department of Psychiatry, First Clinical Medical College/First Hospital of Shanxi Medical University, Taiyuan, China; 2 Shanxi Key Laboratory of Artificial Intelligence Assisted Diagnosis and Treatment for Mental Disorders, First Hospital of Shanxi Medical University, Taiyuan, China; 3 Nursing College, Shanxi Medical University, Taiyuan, China; 4 Drug Clinical Trial Institution, First Hospital of Shanxi Medical University, Taiyuan, China

**Keywords:** blood-brain barrier, glial cells, late-life depression, neuroimmunology, neuroinflammation, single-cell sequencing, spatial multi-omics

## Abstract

Late-life depression (LLD) is a heterogeneous disorder characterised by affective symptoms, cognitive decline, vascular burden, frailty, treatment resistance, and an increased risk of neurodegenerative progression, yet bulk molecular approaches may obscure the cell-type-specific alterations underlying this clinical and biological heterogeneity. This narrative review synthesises single-cell, single-nucleus, spatial, and multi-omic evidence concerning neuronal, glial, vascular, and peripheral immune alterations relevant to LLD, while distinguishing direct findings from clinically defined or older-adult depression cohorts from evidence extrapolated from major depressive disorder, ageing, mild cognitive impairment, Alzheimer’s disease, and experimental models. The strongest current evidence implicates selective excitatory and inhibitory neuronal subtypes and astrocytes in prefrontal affective–cognitive circuit dysfunction, whereas endothelial and neurovascular-unit abnormalities, microglial reprogramming, oligodendrocyte-lineage dysfunction, hippocampal plasticity deficits, and peripheral immune remodelling represent biologically plausible but less well-validated mechanisms. Ageing-related cellular alterations, blood–brain barrier disruption, endothelial–astrocyte dysregulation, neuroinflammation, impaired myelin maintenance, and hippocampal vulnerability may interact to contribute to affective, cognitive, vascular, and treatment-resistant phenotypes. However, direct single-cell and spatial multi-omics evidence from clinically defined LLD cohorts remains scarce, and peripheral immune signatures should be interpreted primarily as accessible systemic correlates rather than direct measures of brain pathology. Overall, current evidence supports a hypothesis-generating multicellular framework rather than validated LLD-specific mechanisms or therapeutic targets. Future studies integrating single-nucleus and spatial multi-omics with longitudinal clinical phenotyping, neuroimaging, peripheral immune profiling, *in situ* validation, and functional perturbation are required to define LLD-specific cellular states, establish causal mechanisms, and identify clinically actionable biomarkers and therapeutic targets.

## 1 Introduction

Late-life depression (LLD) is associated with high rates of disability, recurrence, and treatment resistance (1). Age at onset is clinically relevant: late-onset depression may differ from early-onset depression recurring in later life with respect to genetic vulnerability, cerebrovascular burden, inflammatory profiles, and neurodegenerative trajectories (2). Clinically, LLD often presents as an affective-cognitive-somatic syndrome involving executive dysfunction, apathy, and somatic complaints that overlap with multimorbidity and age-related functional decline, thereby complicating diagnosis (3, 4). LLD is therefore better conceptualised not simply as depression superimposed on ageing, but as a heterogeneous syndrome embedded within brain ageing and associated with cardiovascular disease, frailty, and increased dementia risk (5). Biomarkers such as plasma neurofilament light chain and senescence-related factors suggest axonal injury and chronic inflammation (6, 7), while antidepressant treatment yields suboptimal remission rates and heterogeneous response trajectories (8, 9). This clinical variability may reflect multiple biological substrates, including cerebrovascular pathology, white matter hyperintensities, chronic inflammation, and impaired neuroplasticity (10), challenging unitary models of LLD.

Conventional LLD research has relied largely on peripheral biomarkers, homogenised brain tissue, and bulk RNA sequencing. Although these approaches identify alterations in inflammatory, synaptic, vascular, and neuroplasticity-related pathways, they average signals across mixed cell populations, obscuring their cellular origins and making it difficult to distinguish pathogenic drivers from compensatory responses (11, 12). This limitation is particularly important in LLD, where relevant abnormalities may arise within discrete microglial states (13), hippocampal progenitor or neuronal populations (14), and endothelial or blood-brain barrier components. Bulk profiles cannot determine which cellular populations contribute to affective symptoms, cognitive impairment, vascular dysfunction, or treatment resistance. Reference-based deconvolution using large single-nucleus RNA-sequencing reference panels can partially recover fine-grained cell-type-specific associations from bulk human brain datasets, although its accuracy remains dependent on reference-panel composition and computational assumptions (15).

Single-cell RNA sequencing, single-nucleus RNA sequencing, and single-cell epigenomic approaches resolve disease-associated cell populations and states, capturing transcriptional reprogramming, inflammatory activation, synaptic alterations, and regulatory transitions (16, 17). By localising genetic and pathological signals to specific neuronal, glial, vascular, and immune populations, these technologies can identify cell types preferentially affected by disease risk and ageing-related processes (18, 19). Studies across depression, neurodegeneration, and ageing have implicated selected neuronal and glial subtypes, microglial activation states, oligodendrocyte-lineage cells, and neurovascular components (20, 21). In LLD, these approaches may help link clinical heterogeneity to cellular heterogeneity and determine whether affective symptoms, executive dysfunction, vascular burden, cognitive decline, and treatment resistance arise from distinct or interacting cellular mechanisms. However, single-cell and spatial maps alone do not establish causality and require validation in clinically defined cohorts and functional models.

Direct single-cell and single-nucleus studies of clinically defined LLD remain scarce. Much of the available evidence derives from major depressive disorder, ageing brain atlases, mild cognitive impairment, Alzheimer’s disease, and stress-based animal models. Although these conditions share biological features with LLD, they do not fully reproduce its characteristic cerebrovascular burden, physical multimorbidity, cognitive profile, and treatment resistance. Cross-disorder and cross-species extrapolation must therefore be interpreted cautiously. This review synthesises single-cell, single-nucleus, spatial, and multi-omic evidence on neuronal, glial, vascular, and peripheral immune mechanisms relevant to LLD. Particular attention is given to cross-talk between brain-resident cells and peripheral immune populations, including its modulation by the blood-brain barrier and neurovascular unit, while distinguishing direct LLD evidence from inferential findings. Specifically, we examine which cell types and states are implicated in LLD; how ageing, vascular, inflammatory, and neurodegenerative processes converge at the cellular level; which findings are directly supported and which remain inferential; and how single-cell and spatial multi-omic technologies may inform biomarker discovery, molecular subtyping, and precision interventions.

## 2 A single-cell and spatial multi-omics framework for late-life depression

Single-cell, single-nucleus, and spatial sequencing technologies provide a cell-type-resolved framework for studying depression in older adults. For LLD, their value extends beyond generating detailed molecular catalogues to identifying the cellular populations and states potentially associated with affective symptoms, executive dysfunction, vascular burden, cognitive decline, and treatment resistance. These modalities address complementary questions: transcriptomic approaches identify disease-associated cell types and states; epigenomic approaches reveal regulatory vulnerability; multi-omic approaches integrate regulatory, transcriptional, and protein-level alterations; and spatial approaches place these changes within their anatomical and microenvironmental context.

### 2.1 Single-cell/single-nucleus transcriptomic sequencing

Single-cell RNA sequencing (scRNA-seq) profiles the transcriptomes of individual cells, overcoming bulk averaging and resolving cell-type-specific expression patterns (22). In LLD research, this approach may help determine whether inflammatory, synaptic, vascular, or neuroplasticity-related signals originate from neurons, astrocytes, microglia, oligodendrocyte-lineage cells, endothelial cells, or infiltrating immune populations.

However, dissociation of intact cells from adult brain tissue may induce transcriptional stress and result in the selective loss of vulnerable populations. Single-nucleus RNA sequencing (snRNA-seq) profiles nuclear transcriptomes without requiring viable intact cells and is therefore particularly suitable for frozen post-mortem human brain tissue (23). It reduces many artefacts associated with whole-cell dissociation. By contrast, scRNA-seq generally requires fresher tissue and captures a broader range of cytoplasmic transcripts, whereas snRNA-seq is enriched for nuclear and unspliced transcripts. The two approaches are complementary, but neither alone preserves spatial organisation or captures the temporal dynamics of LLD pathophysiology (24, 25).

These methods can delineate transcriptional alterations across neuronal, glial, vascular, and immune compartments, identify disease-associated subpopulations, and inform molecular subtyping and candidate-target discovery. However, they cannot independently establish causal sequence, distinguish primary pathogenic mechanisms from compensatory responses, or reconstruct spatial tissue organisation. LLD-relevant single-cell and single-nucleus data should therefore be interpreted as cell-type-resolved molecular maps rather than direct evidence of causal pathogenesis.

### 2.2 Single-cell epigenomic sequencing

Transcriptomic profiles represent downstream regulatory outputs and cannot alone reconstruct the epigenetic architecture underlying disease-associated states. Single-cell and single-nucleus assays for transposase-accessible chromatin (ATAC) sequencing, including scATAC-seq and snATAC-seq, map cell-type-specific chromatin accessibility at enhancers, promoters, and transcription-factor binding regions, enabling the inference of relationships between cis-regulatory elements and transcriptional programmes (26).

In LLD, these approaches may help determine whether genetic risk, ageing-related regulatory changes, inflammatory priming, and stress vulnerability converge on particular neuronal, glial, immune, or vascular populations. ATAC-seq can identify regulatory remodelling associated with cell-state transitions, while integration with genome-wide association study (GWAS) data may map disease-risk loci to specific cell types and candidate regulatory elements. However, chromatin accessibility does not necessarily indicate active transcription, and single-cell ATAC-seq data are sparse and analytically demanding.

These limitations have motivated integrative analysis of matched scATAC-seq and scRNA-seq datasets, snATAC-seq and snRNA-seq datasets, and joint single-cell multi-omic assays that measure regulatory and transcriptional states within the same cells (27). In LLD, such frameworks may clarify how ageing, inflammation, vascular burden, and stress reshape the regulatory landscapes of brain and immune cells. However, direct single-cell epigenomic evidence from clinically defined LLD cohorts remains scarce. These methods therefore generate hypotheses concerning regulatory vulnerability but cannot independently establish LLD-specific mechanisms or therapeutic targets.

### 2.3 Single-cell multi-omics and spatial omics technologies

Single-cell transcriptomics alone cannot capture the multilayered molecular architecture of complex disorders. Multi-omic approaches integrate transcriptomic, epigenomic, and proteomic information within the same cells or across matched cell populations. For example, cellular indexing of transcriptomes and epitopes by sequencing (CITE-seq) simultaneously measures RNA transcripts and cell-surface proteins, improving immune-cell annotation beyond transcriptome-based clustering alone (28). In LLD, this may be particularly useful for characterising peripheral immune-cell remodelling, refining immune subtype definitions, and identifying surface-protein phenotypes potentially associated with cognitive decline or treatment response.

Spatial context is also essential for interpreting brain pathology. Spatial transcriptomics preserves tissue architecture and maps or infers the spatial distributions of cellular populations, molecular states, and local intercellular interactions that are lost during tissue dissociation (29). This is particularly relevant to LLD because candidate mechanisms, including neurovascular-unit dysfunction, endothelial-astrocyte signalling, microglial activation, white matter pathology, and hippocampal vulnerability, are spatially organised and depend on local cellular communication. Integration of spatial transcriptomics with single-cell or single-nucleus reference atlases may help localise disease-associated cell states, characterise their interactions with neighbouring cells, and determine whether they are associated with vascular lesions, inflammatory niches, white matter regions, or cognition-related circuits.

Multi-omic and spatial approaches nevertheless have substantial limitations. High dimensionality, data sparsity, batch effects, experimental and computational costs, and incomplete cross-modal alignment complicate integrated analysis of heterogeneous tissues such as the ageing brain. Spatial platforms also involve trade-offs among cellular resolution, tissue coverage, and molecular depth. Beyond these technical limitations, most LLD-relevant spatial and multi-omic evidence currently derives from ageing or neurodegenerative cohorts rather than clinically defined LLD samples (30, 31). These approaches can therefore support anatomical localisation, intercellular communication analysis, and mechanistic hypothesis generation, but they cannot replace longitudinal clinical phenotyping, neuroimaging correlation, neuropathological validation, or functional perturbation experiments. Future LLD-specific spatial transcriptomic studies could address disease-specific limitations by mitigating tissue scarcity through multicentre brain banking and standardised sampling of clinically well-characterised post-mortem tissue. The use of spatial platforms compatible with archived formalin-fixed paraffin-embedded specimens may further expand the availability of suitable human brain tissue (32). Ageing-related molecular variation could be reduced through the inclusion of age-matched controls and by modelling chronological age and vascular or neurodegenerative comorbidities as covariates. Benchmarking LLD-associated signals against lifespan-resolved reference datasets may further help distinguish disease-associated alterations from molecular changes related to normal ageing (33). Integration with single-nucleus reference atlases, neuropathology, neuroimaging, and detailed clinical phenotyping may provide additional support for distinguishing LLD-associated spatial signatures from normal ageing-related molecular changes.

Overall, single-cell and spatial multi-omic technologies can move LLD research from bulk-level associations towards cell-type-resolved mechanistic hypotheses. However, current evidence remains constrained by small samples, cross-sectional and post-mortem designs, incomplete clinical annotation, and limited disease-specific validation. The following sections therefore evaluate available cellular findings while distinguishing directly supported evidence from plausible but unvalidated mechanisms.

## 3 The application and findings of single-cell sequencing technology in late-life depression

Because direct single-cell and single-nucleus evidence from clinically defined LLD cohorts remains limited, this section reviews available findings according to cellular systems and their proximity to LLD pathophysiology. We first discuss older-adult depression-related evidence, and then incorporate findings from major depressive disorder (MDD), ageing brain atlases, mild cognitive impairment (MCI), Alzheimer’s disease (AD), peripheral immune studies, and stress-based models as hypothesis-generating references. Key single-cell, single-nucleus, and spatial multi-omic studies relevant to LLD, together with their evidence levels and major limitations, are summarised in **Table 1**.

**Table 1:** Key single-cell and spatial multi-omic studies relevant to late-life depression.

Evidence tier	Study	Population/model	Technology	Tissue/region	Key cell types	Key findings	Mechanistic axis	Relevance to LLD and limitations
A	Zeng et al. (35)	Older adults from the ROSMAP cohort; antemortem depressive symptoms	snRNA-seq and GWAS integration	DLPFC	Excitatory neurons; inhibitory neurons; astrocytes	Depression-related genetic signals were concentrated in specific neuronal and astrocytic subtypes, with subtype-dependent effects of candidate risk genes.	Synaptic function	Human older-adult brain evidence with direct relevance to LLD-related cellular vulnerability; based on depressive symptoms rather than a clinically defined LLD cohort and limited by its post-mortem, cross-sectional design.
B	Nagy et al. (17)	Human MDD and neurotypical controls	snRNA-seq	DLPFC	Deep-layer excitatory neurons; oligodendrocyte precursor cells	MDD-associated transcriptional changes were concentrated in layer-specific neuronal populations and immature oligodendrocyte-lineage states.	Synaptic function; metabolism	Supports neuronal-glial cell-state dysregulation relevant to LLD; not specific to late-life disease or aging.
B	Chawla et al. (34)	Human MDD and neurotypical controls; 84 donors	snATAC-seq, matched snRNA-seq, genotype and GWAS integration	DLPFC	Deep-layer excitatory neurons; gray-matter microglia	MDD-risk variants and NR4A2-associated regulatory changes were enriched in deep-layer excitatory neurons; microglia showed reduced accessibility at immune-homeostasis transcription-factor binding sites.	Epigenetic regulation; synaptic function; inflammation	Provides a human genetic-to-regulatory-to-cell-type mechanism; lacks LLD and aging-specific stratification.
B	Xiao et al. (54)	Human MDD and controls; 16 male donors	snRNA-seq, snATAC-seq, Visium, DBiT-seq and RNA velocity	Hippocampus	Neural progenitor cells; immature granule neurons; CA neurons; microglia; oligodendrocytes	The atlas identified a neurogenic niche, altered ASCL1 accessibility, increased interferon-inducible genes, reduced TTR expression and CA-neuron transcriptomic instability in MDD.	Neurogenesis; inflammation; epigenetic regulation; metabolism	High-resolution human hippocampal evidence relevant to LLD cognitive and stress phenotypes; male-only cohort and no LLD stratification.
C	Mathys et al. (36)	Human Alzheimer disease and controls	snRNA-seq	DLPFC	Excitatory neurons; SST-positive inhibitory neurons	Specific neuronal subtypes showed selective vulnerability, whereas preservation of selected inhibitory populations was associated with cognitive resilience.	Synaptic function	Provides a reference for cognitive comorbidity and neuronal vulnerability in older adults; AD-specific and not evidence of LLD pathology.
C	Kuan et al. (51)	Human mild cognitive impairment and controls	CITE-seq	Peripheral blood mononuclear cells	Monocytes; T-cell populations	The strongest transcriptomic changes occurred in monocytes and involved inflammatory, innate immune and antigen-presentation pathways.	Inflammation; metabolism	Peripheral reference for cognitive comorbidity and immune remodelling; Non-intracerebral microglia/LLD-specific; WTC male cohort (with mixed exposure histories).
C	Su et al. (56)	Experimental model of impaired adult hippocampal stem-cell homeostasis	scRNA-seq and pseudotime analysis	Dentate gyrus	Neural stem/progenitor cells; granule neurons	Absence of the Klotho+ NSC subpopulation, stem-cell-pool exhaustion and reduced neuronal production were associated with impaired plasticity and depression-like behaviour.	Neurogenesis; synaptic function	Supports a hippocampal neurogenesis mechanism; animal-model evidence without direct validation in LLD.
C	Goodman et al. (58)	Experimental stress model	scRNA-seq	Hippocampus	Microglia; astrocytes	Stress remodelled glial states and cell-cell communication programs linked to inflammatory signalling, metabolic support and neuronal plasticity.	Inflammation; metabolism	Mechanistic reference for stress susceptibility; based on an animal model and not directly generalizable to human LLD.

Evidence tier A denotes human older-adult depression-related brain evidence with direct relevance to LLD; tier B denotes human MDD brain single-cell or multi-omic evidence with mechanistic relevance to LLD; tier C denotes cross-disorder, peripheral, or experimental-model evidence used for mechanistic reference. These tiers reflect disease specificity and translational proximity to LLD rather than methodological quality, causal certainty, or strength of clinical recommendation. Mechanistic axes were standardised across studies as inflammation, synaptic function, neurogenesis, metabolism, and epigenetic regulation.

ROSMAP, Religious Orders Study and Memory and Aging Project; DLPFC, dorsolateral prefrontal cortex; GWAS, genome-wide association study; MDD, major depressive disorder; LLD, late-life depression; OPC, oligodendrocyte precursor cell; SST, somatostatin; CITE-seq, cellular indexing of transcriptomes and epitopes by sequencing; DBiT-seq, deterministic barcoding in tissue for spatial omics; WTC, World Trade Centre.

### 3.1 Prefrontal cortical cell subtypes and the localisation of genetic risk for depression

The dorsolateral prefrontal cortex (DLPFC) regulates emotion, executive function, reward valuation, and cognitive control—domains that are consistently impaired in LLD, manifesting as cognitive decline, apathy, amotivation, and treatment resistance. Bulk transcriptomics has detected synaptic, inflammatory, and neuroplasticity-related alterations in this region, yet these signals reflect population-averaged contributions that obscure cellular origins and may mask disease-relevant subpopulations. snRNA-seq disaggregates these composite signals, enabling the identification of cell-type-specific vulnerabilities in LLD (34).

Zeng et al. profiled the DLPFC from 424 Religious Orders Study and Memory and Aging Project (ROSMAP) participants using snRNA-seq, integrating cell-type-specific transcriptomes with depression GWAS to localise LLD genetic risk to discrete neuronal and glial substrates. Across seven major cell types and over 50 transcriptionally defined subtypes, risk enrichment converged selectively on excitatory neurons, inhibitory neurons, and astrocytes. These data challenge models positing either global brain ageing or a microglia-dominated inflammatory pattern as the primary pathological substrate of LLD (35). LLD risk converges on functional dysregulation of discrete neuronal subtypes, excitatory-inhibitory imbalance, and altered neuron-glial crosstalk. The same risk allele can exert divergent—even opposing—effects across neuronal subtypes: NEGR1 shows inconsistent associations across excitatory and inhibitory subpopulations, indicating that cellular context and age-related circuit remodelling reshape genetic risk penetrance. These findings challenge the reduction of LLD to generic ‘neuronal damage’ or diffuse synaptic dysfunction; instead, they implicate selective, subtype-specific cellular vulnerabilities. Candidate risk genes—including TAOK3, MADD, SCAI, CHUK, and ANKRD36—converge on pathways governing neuronal excitability (KCNN2), synaptic architecture (WASF3), and cytokine signalling (SOCS6), linking genetic susceptibility to discrete neurobiological mechanisms. Critically, after accounting for AD pathology and cognitive decline, LLD-associated signalling persists, arguing against its interpretation as mere ‘ageing noise’ and supporting a model in which genetic susceptibility interacts with subtype-specific circuit abnormalities to produce the LLD phenotype.

Complementary evidence from MDD further refines the regulatory architecture of depression risk. A multi-omic profiling of the DLPFC in MDD integrated single-nucleus ATAC sequencing (snATAC-seq), snRNA-seq, and genetic variant data across over 200,000 nuclei, achieving the resolution necessary to link cis-regulatory variation to cell-type-specific transcriptomic states (34). MDD-associated variants were enriched in open chromatin regions of deep-layer excitatory neurons, disrupting transcription factor binding sites implicated in synaptic signalling—notably the stress-responsive factor NR4A2. A discrete microglial subpopulation concurrently showed reduced chromatin accessibility at immune homeostasis regulatory elements, implicating these cells in neuroimmune dysregulation. These findings establish a multi-omic framework mapping MDD risk from genetic variation through epigenetic regulation to cell-type-specific vulnerability. Direct extrapolation to LLD is complicated, however, by the distinct cerebrovascular, inflammatory, and neurodegenerative ageing trajectories of late-onset depression, which may reshape chromatin accessibility independently of MDD-associated genetic architecture.

AD and cognitive impairment studies converge on a similar principle of subtype selectivity. Mathys et al. showed that AD-related pathology concentrates in deep-layer excitatory and somatostatin-positive inhibitory neurons; preservation of specific inhibitory subtypes correlates with cognitive resilience (36). Cain et al. implicated oligodendrocyte and broader inhibitory neuron dysfunction in cognitive decline (37). These patterns are not LLD-specific, yet they establish that ageing-related cognitive and affective disturbances involve selective cellular vulnerabilities and cross-population network dysfunction rather than diffuse neuronal loss. Their relevance to LLD depends on whether shared risk factors—vascular insult, chronic inflammation, or APOE ϵ4—converge on the same subtypes in depressed older adults.

Direct LLD evidence implicates excitatory and inhibitory neuronal subtypes and astrocytes as primary cellular substrates of genetic risk. AD-derived findings offer circumstantial support at best; their relevance to LLD hinges on whether ageing-related cognitive decline and affective disturbance share convergent cellular vulnerabilities or merely represent overlapping end-stage phenotypes. Resolving this question will require LLD-specific brain banking, spatial transcriptomic validation, and functional assays that can distinguish primary pathogenic mechanisms from secondary or compensatory changes.

### 3.2 Ageing-related cellular states and their multicellular effects

LLD unfolds within a systemic ageing milieu characterised by cellular senescence, chronic low-grade inflammation, mitochondrial dysfunction, proteostatic imbalance, and attenuated tissue repair. Unlike depression in younger adults, LLD clusters with cerebrovascular disease, cognitive decline, frailty, and neurodegenerative trajectories—indicating that its mechanisms extend beyond limbic circuit dysfunction to encompass brain-wide cellular state alterations. Single-cell and single-nucleus transcriptomics can resolve this heterogeneity at the resolution of individual cell types and states, yet ageing-related transcriptional signatures overlap with stress, inflammation, and neurodegeneration, complicating their attribution to chronological ageing versus disease-related acceleration. Ageing-related gene set enrichment, pseudotime trajectory inference, and intercellular communication mapping can localise senescence signals to specific cell types and discriminate compositional shifts from intrinsic transcriptional reprogramming. However, current ageing clocks in single-cell data are largely trained on peripheral tissues, and their transferability to the ageing brain—and specifically to LLD—remains uncertain.

Lian et al. integrated bulk transcriptomic data from 144 patients with MDD and 72 healthy controls with single-cell RNA-sequencing data to examine the distribution and potential functions of senescence-related genes (38). Their bulk analysis identified 33 senescence-related differentially expressed genes and showed significantly higher senescence-related gene-set scores in MDD. At the single-cell level, 19 cellular clusters were identified, including neuronal, glial, vascular, and immune populations, with endothelial cells exhibiting the highest senescence-related gene scores. Cell-cell communication analysis further predicted stronger astrocytic responses to signals from high-senescence-score endothelial cells, accompanied by activation of Notch-related communication, particularly the DLL4-NOTCH1 and ADAM17-NOTCH1 axes. These findings suggest that senescence-associated alterations in depression may be cell-type selective and may extend beyond cell-autonomous dysfunction through endothelial–astrocyte communication. However, because the study examined MDD rather than clinically defined LLD and did not provide age-specific stratification, its relevance to LLD remains inferential.

More broadly, this cell-type-selective pattern supports the possibility that multiple neuronal, glial, vascular, and immune populations deviate from homeostasis along distinct trajectories rather than undergoing a uniform, brain-wide senescence programme. These altered states may propagate beyond cell-autonomous boundaries through the senescence-associated secretory phenotype (SASP), inflammatory mediators, oxidative stress, mitochondrial dysfunction, and disrupted intercellular signalling (39). Notably, elevated SASP-related molecular signatures have been reported in patients with LLD, supporting a potential upstream role of cellular senescence in LLD-associated multicellular dysfunction (40). However, the causal contribution of cellular senescence and its mechanistic relationship with chronic stress and vascular injury require further investigation. Moreover, gene-set scores do not establish irreversible cellular senescence, and predicted ligand-receptor interactions do not demonstrate functional communication. LLD-specific longitudinal studies combining spatial transcriptomics, cell-cycle arrest and DNA-damage markers, SASP profiling, *in situ* validation, and functional perturbation are therefore needed to distinguish normal ageing from pathological acceleration and clarify its effects on neurovascular, neuroimmune, and hippocampal systems.

### 3.3 Neurovascular-unit dysfunction: endothelial and astrocytic alterations

Vascular pathology is a prominent feature of LLD, which is associated with increased cerebrovascular burden, white matter hyperintensities (WMH), microcirculatory dysfunction, and accelerated cognitive decline (41). The vascular depression hypothesis proposes that ischaemic or hypoperfusive lesions disrupt frontostriatal circuits and white matter tracts, thereby contributing to depressive phenotypes. However, the cellular and molecular substrates underlying this macroscopic model—particularly the vascular and glial populations involved in circuit dysfunction—remain incompletely understood. Single-cell and single-nucleus transcriptomics may help resolve these vascular–cognitive–affective associations at cell-type resolution.

Within the neurovascular unit, microvascular endothelial cells form the core of the blood–brain barrier (BBB), whereas astrocytes contribute to BBB maintenance, neurotransmitter homeostasis, metabolic support, and neurovascular coupling. Integrating transcriptomic profiles with ageing-related gene signatures and ligand–receptor analyses can localise altered programmes to specific endothelial or pericyte subtypes and infer their communication with astrocytes, microglia, and neurons (42). Lian et al. identified pronounced ageing-related signatures in cerebrovascular endothelial cells (38). Endothelial populations with elevated senescence scores were predicted to communicate with astrocytes through DLL4–NOTCH1 and ADAM17–NOTCH1 axes, implicating altered NOTCH-related signalling in neurovascular ageing. Endothelial dysfunction may compromise BBB integrity, cerebral perfusion, and microcirculation, thereby increasing the influence of peripheral inflammatory signals on the brain (43, 44). Reactive astrocytes may further impair glutamate homeostasis, metabolic support, and neurovascular coupling, increasing the vulnerability of prefrontal emotional and executive networks. Endothelial senescence, BBB disruption, and endothelial–astrocyte dysregulation therefore represent plausible mechanisms for a vascular subtype of LLD (45). Integrating molecular biomarkers with WMH burden, cerebral perfusion indices, and executive-function measures may help refine vascular LLD subtyping and treatment-response prediction.

These findings nevertheless require cautious interpretation. The “high-senescence” phenotype is primarily based on gene-set enrichment scores rather than direct evidence of irreversible cellular senescence (46), while the proposed intercellular communication pathways are computationally inferred from ligand–receptor expression rather than empirically demonstrated. Their biological validity requires spatial transcriptomic localisation, *in situ* protein detection, and functional perturbation assays. It also remains unclear whether endothelial and astrocytic alterations are reversible disease-related states, fixed consequences of ageing, or secondary responses to vascular and inflammatory injury.

Overall, neurovascular-unit dysfunction may provide a cellular link between cerebrovascular burden, BBB impairment, neuroinflammation, and emotional–executive network dysfunction in LLD. However, whether this vascular–neuroimmune axis is causal, compensatory, or epiphenomenal, and whether it acts upstream or downstream of depressive pathogenesis, remains unresolved and requires validation in longitudinal, interventional, and clinically defined LLD cohorts.

### 3.4 Heterogeneity of microglial states and neuroimmune imbalance

Microglia mediate immune surveillance, synaptic pruning, debris clearance, and neural circuit homeostasis. Single-cell and single-nucleus transcriptomics have resolved microglia into transcriptionally distinct states—homeostatic, pro-inflammatory, interferon-responsive, phagocytic, and senescent—whose relative proportions shift with age, brain region, stress exposure, and disease stage (47). These state transitions are inferred from snapshot transcriptomic profiles and may not capture the full temporal dynamics of microglial activation *in vivo*. In LLD, state heterogeneity may account for variable neuroinflammatory burden, cognitive trajectories, and treatment resistance, though whether specific microglial states are causal drivers or secondary responders remains unresolved.

Ageing and chronic stress converge to shift microglia from homeostatic surveillance to pro-inflammatory, dysfunctional states. In rodent stress models, prolonged glucocorticoid exposure reprogrammes microglial transcriptional and epigenetic programmes, upregulating inflammatory, interferon, phagocytic, and antigen-presentation signatures (48). Senescent microglia concurrently display heightened inflammatory signalling alongside impaired debris clearance, fostering the accumulation of damaged synapses, myelin debris, and protein aggregates that sustain chronic neuroimmune activation (49). Whether stress-induced microglial reprogramming in rodents recapitulates the slower, vascular-inflammatory trajectory of human LLD remains uncertain; the translational gap between acute stress paradigms and late-onset depressive pathogenesis is substantial. Microglial states resist binary classification as ‘pro-inflammatory’ or ‘anti-inflammatory’; their functional impact is context-dependent, shifting between pathogenic, homeostatic, and compensatory roles across disease stages. Such plasticity complicates the attribution of specific transcriptomic signatures to discrete functional outcomes.

Activated microglia release interleukin-1β (IL-1β), tumour necrosis factor-α (TNF-α), and interleukin-6 (IL-6), directly modulating neuronal excitability, neurotransmitter metabolism, and synaptic plasticity. Complement-mediated or phagocytic dysregulation disrupts synaptic pruning homeostasis, compromising prefrontal–hippocampal circuit integrity. Whether these cytokine-driven changes are reversible or represent fixed circuit damage has direct implications for therapeutic windows. Microglial signalling further drives astrocyte reactivity and impairs oligodendrocyte precursor cell-mediated myelin maintenance. Blood–brain barrier disruption and peripheral immune infiltration amplify these neuroinflammatory cascades. Yet the microglial subsets implicated in these circuits have been characterised primarily in Alzheimer’s disease; their direct relevance to LLD pathophysiology remains inferential (50). Distinguishing AD-like microglial activation from LLD-specific neuroinflammatory trajectories will require disease-specific single-cell profiling.

Peripheral blood mononuclear cells (PBMCs) in MCI show inflammatory, antigen-presenting, and immunosenescent shifts in monocyte and lymphocyte subsets (51, 52). Yet these peripheral signatures do not recapitulate brain-resident glial states; the blood–brain barrier, divergent cellular ontogeny, and distinct microenvironmental cues dissociate systemic immune markers from central neuroinflammatory dynamics.

Microglia constitute a putative neuroimmune hub integrating ageing, stress, peripheral immune signals, synaptic pathology, and white matter injury. Yet single-cell microglial atlases in LLD remain sparse, and whether rodent stress models or AD cohorts recapitulate the vascular-inflammatory trajectory of human LLD is unresolved. Whether interferon-responsive microglia track with WMH burden and executive decline—and whether they are druggable targets or epiphenomenal markers—awaits prospective validation.

### 3.5 The potential role of hippocampal maturity and age-related cellular states in the depressive phenotype

The hippocampus mediates memory consolidation, contextual encoding, emotional regulation, and negative feedback control of the hypothalamic-pituitary-adrenal (HPA) axis. Hippocampal dysfunction in LLD manifests as episodic memory decline, comorbid anxiety, and attenuated stress recovery. Depression has been linked to hippocampal atrophy, reduced neurogenesis, and HPA axis dysregulation (53), yet bulk neuroimaging and histology cannot resolve which cell types drive these macroscopic changes. Single-cell and single-nucleus transcriptomics can help distinguish hippocampal ageing from depression-related pathology by mapping shifts in neural stem cells, granule neurons, astrocytes, and microglia. However, LLD-specific hippocampal single-cell evidence remains scarce, and current mechanistic insights are derived primarily from human MDD studies, ageing-related neurogenesis research, and experimental stress or neuropsychiatric models. Their applicability to LLD remains uncertain because vascular and inflammatory comorbidities may independently reshape the hippocampal microenvironment.

A multi-omic atlas of the adult hippocampus in MDD—integrating snRNA-seq, snATAC-seq, and spatial transcriptomics using Visium and deterministic barcoding in tissue for spatial omics (DBiT-seq)—profiled over 290,000 nuclei and identified neural progenitor cells (NPCs) and immature granule neurons as the primary cellular substrates of MDD-related hippocampal dysfunction (54). These progenitor populations displayed aberrant chromatin accessibility at neurodifferentiation loci, including ASCL1, alongside transcriptional dysregulation, indicating disrupted neurogenic lineage commitment. RNA velocity analysis further revealed destabilised maturation trajectories in cornu Ammonis (CA) pyramidal neurons. Interferon signature upregulation and reduced transthyretin (TTR) expression implicated concurrent neuroinflammation and impaired thyroid hormone transport as convergent pathogenic mechanisms. Among the MDD-associated hippocampal alterations identified, NPC vulnerability and interferon-signature upregulation may provide mechanistic context relevant to depression-related hippocampal dysfunction. However, these findings were derived from an MDD cohort without age-specific stratification and therefore should not be interpreted as direct evidence for LLD. Their relevance to LLD requires disease-specific validation, particularly given the marked suppression of adult neurogenesis in the ageing human hippocampus and the potential effects of vascular and inflammatory comorbidities on progenitor niches.

Hagihara et al. (55) integrated hippocampal transcriptomic and single-cell data across rodent neuropsychiatric models, identifying ‘overmaturation’ or ‘accelerated ageing’-like expression signatures alongside synaptic pathway alterations and anxiety-like behaviour. Inflammation-driven models mapped these signatures primarily to microglia, whereas glucocorticoid stress models implicated astrocytes and mature granule cells—indicating that convergent hippocampal ageing phenotypes can arise from divergent cellular mechanisms. These animal patterns diverged from bulk transcriptomic profiles in human psychiatric disorders: MDD cohorts showed either accelerated ageing, pseudo-immaturity, or negative ageing correlations. Abnormal hippocampal maturity thus appears to be a cross-diagnostic, aetiologically heterogeneous transcriptional state rather than an LLD-specific biomarker. Whether vascular and inflammatory comorbidities in LLD independently remodel hippocampal progenitor niches—beyond the stress and neuroinflammatory trajectories captured in these models—remains unresolved.

Animal snRNA-seq and pseudotime analyses reveal that disruption of adult hippocampal neural stem cell (NSC) homeostasis depletes specific stem cell subpopulations, attenuates neurogenesis, and impairs synaptic plasticity—changes that track with anxiety- and depression-like behaviour (56). Ageing dentate gyrus atlases further show that senescence spans the entire neurogenic trajectory, from quiescent NSC activation through fate commitment to newborn neuron maturation, with local immune infiltration imposing additional regenerative constraints (57). Stress models implicate microglial and astrocytic state remodelling via inflammatory mediators, metabolic support deficits, and altered intercellular communication in granule cell dysfunction and hippocampal circuit plasticity impairment (58). Yet these models capture acute or subchronic stress rather than the chronic, low-grade inflammatory and vascular insults that characterise LLD, and their relevance to the markedly suppressed adult neurogenesis observed in ageing humans is uncertain. Depletion of neural stem/progenitor cells (NSPCs) and impaired granule cell generation constrain adult hippocampal neurogenesis and dentate gyrus plasticity (59). Astrocytic dysfunction disrupts glutamate clearance, metabolic support, and synaptic homeostasis, whilst microglial inflammatory reprogramming and aberrant phagocytosis damage hippocampal circuits via cytokine release and excessive synaptic pruning (60). These convergent mechanisms—primarily characterised in animal stress models and MDD—plausibly bridge ageing, chronic stress, and depression-related cognitive-emotional phenotypes. Their direct operation in LLD, where vascular and inflammatory comorbidities may independently impair hippocampal niches, awaits disease-specific validation.

Hippocampal ageing, as resolved by single-cell atlases, converges on impaired neurogenesis, attenuated granule cell plasticity, and glial state reorganisation—reflecting a multicellular network disturbance rather than unitary cell loss. Yet these atlases derive predominantly from animal models, ageing brain cohorts, and cross-disease comparisons; LLD-specific hippocampal single-cell data remain sparse. Prospective studies integrating single-nucleus transcriptomics, spatial transcriptomics, hippocampal imaging, and longitudinal cognitive phenotyping within clinically annotated LLD cohorts are needed to test whether these cellular states predict stress vulnerability, cognitive trajectories, and treatment resistance.

Taken together, available single-cell evidence suggests that LLD involves selective abnormalities across neuronal, glial, vascular, and hippocampal systems rather than diffuse brain-wide dysfunction. These alterations may jointly link affective symptoms, executive dysfunction, vascular burden, cognitive decline, and treatment resistance to multicellular network disruption. However, their evidential strength varies substantially, with only a limited subset supported directly by clinically defined LLD cohorts. The following section therefore evaluates these findings according to their disease specificity and level of evidence.

## 4 Evidence synthesis and inferential boundaries in late-life depression

### 4.1 Similarities and differences between late-life depression and related disease models

Because direct single-cell evidence from clinically defined LLD cohorts remains limited, evidence from MDD, ageing brain atlases, MCI, AD, peripheral immune studies, and experimental stress models is used in this review as complementary and hypothesis-generating support. These disease contexts are relevant to LLD because they share several biological features, including affective disturbance, cognitive impairment, synaptic dysfunction, neuroinflammatory activation, glial reprogramming, vascular injury, and ageing-related cellular vulnerability. In particular, MDD provides the closest reference for depression-related prefrontal, hippocampal, synaptic, and stress-associated mechanisms (17, 31, 34); AD and MCI provide useful models for neuronal vulnerability, cognitive decline, microglial activation, and neurovascular dysfunction in the ageing brain (36, 37, 50–52); ageing atlases define the cellular background in which LLD emerges (38, 42); and stress-based animal models offer experimentally tractable evidence for neuroimmune and glial mechanisms (48, 55–58).

However, these similarities should not obscure important differences. MDD does not fully reproduce the cerebrovascular burden, frailty, multimorbidity, white matter abnormalities, cognitive decline, and treatment resistance that characterise LLD (2, 5, 8, 10, 41). Vascular comorbidities may further distinguish the cellular landscape of LLD from that observed in MDD or AD. In LLD, chronic small-vessel disease, cerebral hypoperfusion, and WMH burden may preferentially affect endothelial cells, pericytes, astrocytes, and oligodendroglial populations, potentially enhancing BBB dysfunction, neurovascular uncoupling, inflammatory signalling, and myelin vulnerability (41–46). By contrast, MDD single-cell studies have predominantly highlighted neuronal, synaptic, and oligodendroglial alterations without specific stratification for age-related vascular burden (17, 34). AD and neurodegenerative forms of MCI are primarily associated with processes such as amyloid deposition, tau pathology, protein aggregation, and progressive neuronal loss, which strongly shape their associated cellular states (36, 37, 50–52). LLD, in contrast, is clinically characterised by affective, motivational, and cognitive symptoms, often accompanied by variable treatment responses, and may occur with or without overt dementia pathology. Thus, vascular comorbidity in LLD may superimpose a neurovascular and white-matter-related transcriptional component on depression-associated cellular alterations, although this possibility remains to be directly tested in LLD cohorts integrating single-cell profiling with WMH burden and cerebral perfusion measures. Ageing-related cellular states may represent normal senescence, adaptive stress responses, or disease-associated acceleration, and therefore cannot be equated with LLD-specific mechanisms (38, 39). Similarly, animal stress models provide mechanistic insight but often fail to capture the chronic vascular, inflammatory, and multimorbid context of human LLD (48, 55–58).

Therefore, cross-disorder evidence is useful for establishing biological plausibility, but disease-specific conclusions should be restricted to findings replicated in clinically annotated LLD cohorts.

### 4.2 Cell-type-based synthesis and evidence hierarchy

The evidence reviewed above supports a multicellular model of LLD, but the strength of evidence differs substantially across cell types. At present, the most direct support comes from prefrontal neuronal and astrocytic alterations identified in older-adult depression-related datasets. Excitatory and inhibitory neuronal subtypes appear to be linked to depression-related genetic vulnerability, while astrocytes may participate in altered neuron-glia communication and affective-cognitive circuit dysfunction. These findings provide the strongest current basis for a cell-type-specific model of LLD (35).

Endothelial cells, pericytes, astrocytes, and other neurovascular-unit components represent a second major candidate axis. The evidence is clinically plausible because vascular burden, WMH, BBB dysfunction, and executive impairment are central to LLD. However, most single-cell evidence for endothelial senescence and endothelial-astrocyte signalling is still derived from ageing or neurodegenerative cohorts. Thus, neurovascular dysfunction should be considered a highly plausible but incompletely validated mechanism for vascular LLD (41–46).

Microglial and peripheral immune mechanisms constitute a third axis. These findings are supported by convergent evidence from MDD, AD, MCI, ageing, peripheral immune studies, and stress models, but direct LLD brain evidence remains limited. Microglial inflammatory, interferon-responsive, phagocytic, or senescent states may contribute to synaptic pruning abnormalities, white matter injury, and treatment resistance. Nevertheless, because AD-related microglial activation may reflect amyloid and tau pathology, and peripheral immune signatures do not directly recapitulate brain-resident microglia, these mechanisms should be framed as inferred rather than definitive (47–52).

Oligodendrocyte-lineage and hippocampal progenitor/neurogenic mechanisms are also relevant but currently more speculative in LLD. Alterations in oligodendrocytes and oligodendrocyte precursor cells (OPCs) may help explain white matter abnormalities and psychomotor or cognitive symptoms, while hippocampal progenitor, granule neuron, astrocytic, and microglial changes may contribute to memory impairment and stress vulnerability. However, these conclusions rely heavily on MDD, AD, ageing, and animal studies. Their direct contribution to LLD requires validation in clinically annotated older-adult cohorts (17, 54–60).

Overall, current evidence supports a graded model: prefrontal neuronal and astrocytic vulnerability is the most directly supported axis; neurovascular and microglial mechanisms are biologically plausible but incompletely validated; hippocampal neurogenic and oligodendroglial mechanisms remain hypothesis-generating; and peripheral immune signatures should be treated as accessible systemic correlates rather than direct measures of brain pathology. This hierarchy helps preserve the value of cross-disease inference while avoiding overstatement of LLD-specific conclusions. These findings are integrated into a hypothesis-generating multicellular network model linking the clinical context of LLD with cell-type-specific mechanisms and disease outcomes (**Figure 1**).

**Figure 1:**
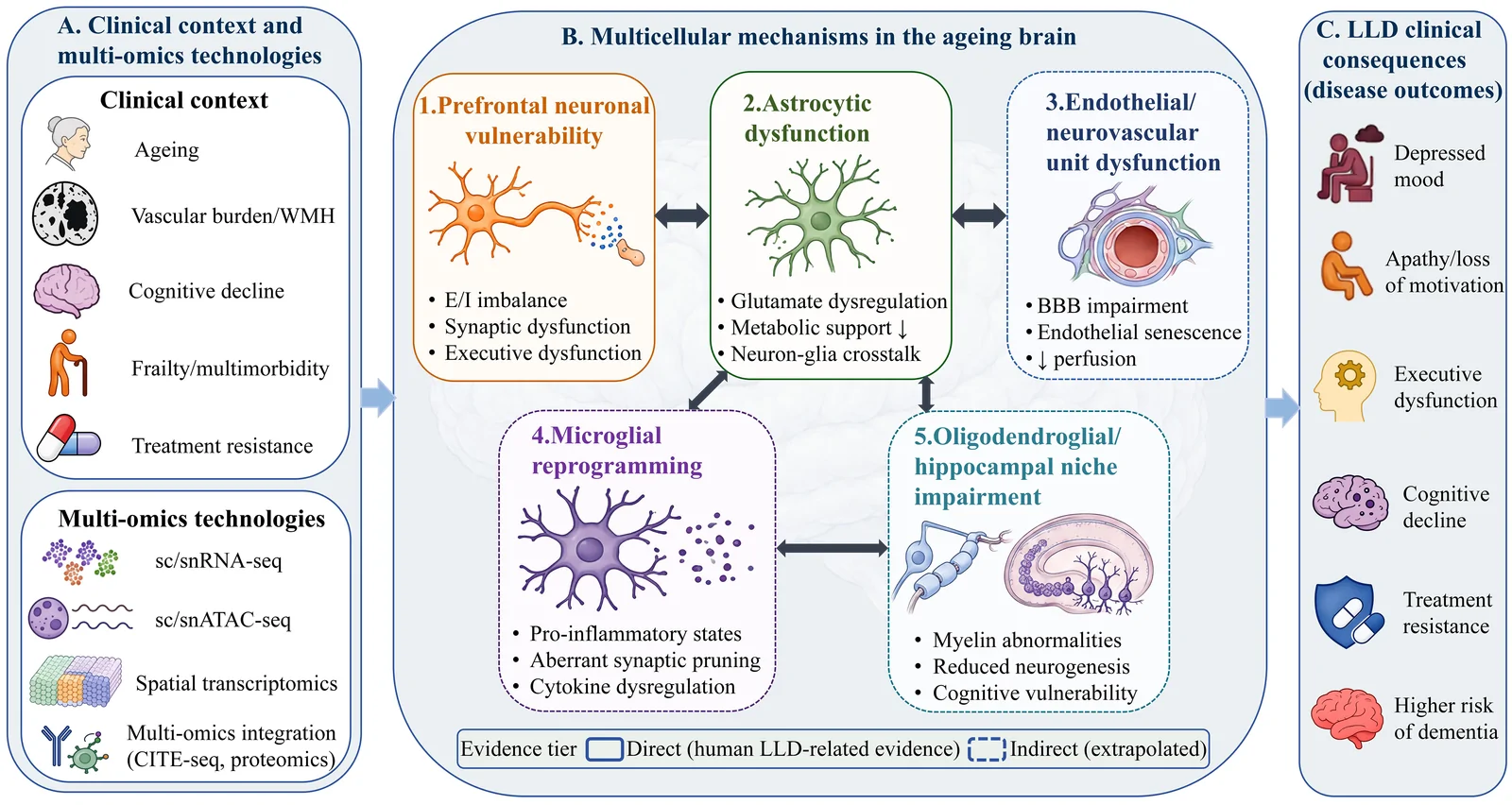
A multicellular network model of late-life depression informed by single-cell and spatial multi-omics. This schematic illustrates how clinical vulnerability factors and multi-omics technologies may converge to define cellular mechanisms and clinical outcomes in late-life depression (LLD). Clinical contexts include ageing, vascular burden or white matter hyperintensities, cognitive decline, frailty or multimorbidity, and treatment resistance. Single-cell, single-nucleus, spatial, and integrated multi-omics approaches may identify alterations in prefrontal neurons, astrocytes, endothelial or neurovascular cells, microglia, and oligodendroglial or hippocampal niches. Solid boxes indicate mechanisms supported by human evidence with direct relevance to LLD, whereas dashed boxes indicate extrapolated or indirect evidence. The final component of the schematic depicts the proposed clinical outcomes of these multicellular alterations, including depressed mood, apathy, executive dysfunction, cognitive decline, treatment resistance, and increased dementia risk. Overall, the model summarises the proposed links among clinical vulnerability factors, multicellular mechanisms, and disease outcomes, and provides a hypothesis-generating framework for molecular subtyping, biomarker discovery, cell-type-specific target identification, and personalised stratification. BBB, blood–brain barrier; CITE-seq, cellular indexing of transcriptomes and epitopes by sequencing; E/I, excitatory/inhibitory; LLD, late-life depression; sc/snATAC-seq, single-cell/single-nucleus assay for transposase-accessible chromatin using sequencing; sc/snRNA-seq, single-cell/single-nucleus RNA sequencing; WMH, white matter hyperintensities.

## 5 From single-cell discoveries to potential therapeutic targets and precision interventions

Single-cell and spatial multi-omic studies provide a framework for translating LLD-related cellular heterogeneity into potential intervention strategies. Rather than focusing solely on monoaminergic pathways traditionally implicated in depression, this framework broadens the mechanistic perspective by resolving cell-type-specific vulnerabilities and intercellular interactions, thereby identifying potential therapeutic targets involving neuronal and astrocytic circuits, neurovascular-unit dysfunction, microglial and peripheral immune activation, oligodendroglial/myelin impairment, and hippocampal plasticity deficits. However, most candidate targets remain inferential and require validation in clinically annotated LLD cohorts, functional perturbation models, and prospective treatment studies. The following section therefore considers how single-cell evidence may inform target identification, drug repositioning, and precision stratification while avoiding premature claims of LLD-specific therapeutic mechanisms.

### 5.1 Identification of cell-type-specific targets

Single-nucleus transcriptomics disaggregates bulk-averaged signals, resolving disease-susceptible subpopulations and their molecular networks. Nagy et al. profiled the DLPFC in MDD using snRNA-seq, identifying transcriptional dysregulation in deep-layer excitatory neurons and immature OPCs (17). These MDD-derived cellular substrates—impaired neuronal excitability, synaptic plasticity, and oligodendroglial support—plausibly track with the executive dysfunction, psychomotor retardation, and white matter abnormalities seen in LLD, though their direct relevance to late-onset vascular and inflammatory trajectories remains inferential. Subtype-specific expression of KCNN2, GRIA3, and KCNK2 implicates ion channel dysfunction, glutamatergic signalling, and synaptic remodelling in LLD-relevant circuit pathology (61). OPC differentiation, myelination, and axonal metabolic support are candidate mechanisms for LLD-related white matter damage. Xie et al. reported OPC transcriptional signatures with discriminative value in MDD (62); whether MALAT1 or related long non-coding RNAs (lncRNAs) drive oligodendroglial dysfunction in LLD—or merely correlate with disease state—awaits mechanistic validation. Cathomas et al. reported elevated matrix metalloproteinase-8 (MMP8) in peripheral and brain-infiltrating monocytes following chronic stress, with parallel serum elevations in MDD (63). Whether this myeloid axis translates to LLD—where vascular comorbidities may independently modulate monocyte trafficking—remains unresolved. Li et al. identified cell-type-specific metabolic disruption in the MDD prefrontal cortex, with impaired oxidative phosphorylation and glucose utilisation (64). These profiles may not recapitulate the mitochondrial dysfunction accompanying ageing and vascular pathology in LLD. Single-cell atlases have shifted LLD targets from diffuse monoamine hypotheses to discrete neuronal subtypes, oligodendroglial lineages, myeloid immunity, and metabolic networks. Yet these targets remain largely inferred from MDD, ageing, and stress models; their direct validation in LLD cohorts—and functional confirmation that they are druggable rather than epiphenomenal—awaits prospective study.

### 5.2 Drug repositioning and personalised treatment strategies

Single-cell and single-nucleus transcriptomics guide drug repurposing by mapping disease-associated subpopulations to druggable molecular nodes and integrating these signatures with pharmacological perturbation databases to identify compounds that reverse aberrant expression or restore network homeostasis. This cell-type-resolved strategy outperforms bulk-deconvoluted approaches in stratifying patient heterogeneity and matching cellular pathologies to targeted interventions (65, 66). Its translation to LLD, however, is constrained by the scarcity of disease-specific atlases, uncertain drug penetration across the ageing BBB, and the lack of validated platforms for cell-type-specific pharmacodynamic readouts in clinically relevant cohorts. LLD likely reflects convergent age-related pathologies rather than a unitary disease mechanism. Proposed molecular subtypes include neuroinflammatory (67, 68), involving peripheral inflammation and microglial activation; vascular-BBB (69), involving vascular risk and WMH burden; synaptic, involving glutamate dysregulation and neuron-glial dysfunction; oligodendroglial-myelin (70), involving impaired myelin homeostasis; and metabolic-senescent, involving mitochondrial and energy dysfunction. These subtypes are not mutually exclusive and frequently co-occur in the ageing brain. Single-cell and single-nucleus transcriptomics can map these pathologies to discrete cell states, yet their utility for LLD molecular subtyping is constrained by cross-sectional designs, limited sample sizes, and lack of prospective validation. This taxonomy remains provisional.

Precision intervention hinges on multimodal biomarkers. Single-cell and single-nucleus transcriptomic signatures must be integrated with peripheral inflammatory and metabolic profiles, neuroimaging, cognitive phenotyping, vascular risk stratification, frailty indices, and pharmacogenomic data to build clinically predictive models. A feasible translational framework could use LLD-specific single-cell and spatial datasets as mechanistic reference maps to define disease-associated cellular states. Because repeated brain-tissue profiling is not feasible in routine clinical settings, these cellular states would primarily serve as mechanistic anchors that could be linked to more accessible peripheral, neuroimaging, and clinical markers in real-world cohorts. These multimodal features could then be integrated with clinically relevant dimensions, including cognitive impairment, vascular burden, frailty, inflammatory or metabolic profiles, and treatment response, to identify reproducible patient subgroups. Clinical, cognitive, and structural magnetic resonance imaging (MRI) features already predict treatment outcomes in LLD (71). T-cell inflammatory states correlate with antidepressant response in MDD (72); whether these peripheral immune signatures or their brain-resident microglial counterparts predict LLD treatment trajectories remains untested. Prospective studies are therefore needed to determine the stability of these multimodal subtypes and their ability to predict treatment response, relapse, cognitive trajectories, and functional recovery, and to establish whether single-cell-informed biomarkers provide clinically actionable stratification beyond traditional predictors.

## 6 Conclusions

Current single-cell and spatial multi-omic evidence supports a hypothesis-generating multicellular model of LLD involving selective neuronal vulnerability, astrocytic dysfunction, microglial reprogramming, neurovascular impairment, disrupted myelin maintenance, hippocampal plasticity deficits, and peripheral immune remodelling. However, direct evidence from clinically defined LLD cohorts remains scarce, and many proposed mechanisms are extrapolated from MDD, ageing, MCI/AD, or experimental models. Spatial multi-omic research is particularly limited by the paucity of LLD-specific tissue datasets, trade-offs among spatial resolution, molecular depth, and tissue coverage, and incomplete cross-modal integration. Existing studies are also constrained by small samples, cross-sectional post-mortem designs, incomplete clinical annotation, and limited causal validation. Future studies should integrate longitudinal LLD cohorts with single-nucleus, spatial, epigenomic, proteomic, neuroimaging, and peripheral immune data, alongside *in situ* validation and functional perturbation, to define LLD-specific cellular states, spatial interactions, molecular subtypes, biomarkers, and precision interventions.
